# Molecular identification of potential leishmaniasis vector species within the *Phlebotomus* (*Euphlebotomus) argentipes* species complex in Sri Lanka

**DOI:** 10.1186/1756-3305-6-302

**Published:** 2013-10-18

**Authors:** Kanapathy Gajapathy, Lalanthika BS Peiris, Sara L Goodacre, Anjana Silva, Pavilupillai J Jude, Sinnathamby N Surendran

**Affiliations:** 1 Department of Zoology, Faculty of Science, University of Jaffna, Jaffna 40000, Sri Lanka; 2 Regional Office, Anti Malaria Campaign, Hambantota 82000, Sri Lanka; 3 School of Biology, University of Nottingham, Nottingham NG7 2RD, UK; 4 Department of Parasitology, Faculty of Medicine and Allied Sciences, Rajarata University of Sri Lanka, Anuradhapura 50008, Sri Lanka

**Keywords:** Argentipes complex, Leishmaniasis, Sibling species, Sri Lanka, Vector

## Abstract

**Background:**

Leishmaniasis is an emerging vector-borne disease in Sri Lanka. *Phlebotomus* (*Euphlebotomus*) *argentipes* sensu lato Annandale and Brunette 1908 is suspected to be a potential vector. Three sibling species have been reported in the species complex based on analysis of morphological data. A study was carried out in different parts of Sri Lanka including cutaneous leishmaniasis prevailing localities to characterise the sibling species of *Phlebotomus* (*Euphlebotomus*) *argentipes* sensu lato and to establish their possible role in *Leishmania* transmission.

**Methods:**

Sandflies were collected using cattle baited trap nets and mouth aspirator. They were identified based on existing taxonomic keys. Sequences of amplified cytochrome oxidase subunit I (CO I), cytochrome oxidase b (cyt b), internal transcribed spacer 2 (ITS2), 18s and 28s rDNA regions were analysed to confirm the number of sibling species. Vectorial capacity of the sibling species was checked by detecting human and *Leishmania* DNA.

**Results:**

Sandflies collected using different techniques were processed for identification, parasite detection and molecular characterization. The 18s, 28s rDNA and cytochrome oxidase subunit I (CO I), internal transcribed spacer 2 (ITS2) and cytochrome b oxidase (cytb) sequences confirmed that the species belonged to the Argentipes complex. 18s and 28s sequences did not show any variation among the proposed sibling species. The phylogeny created from mitochondrial CO I and cytochrome b data and from the nuclear ITS2 region supports the existence of only two groups of flies (termed A and B) from *Phlebotomus* (*Euphlebotomus*) *argentipes* complex instead of the previously proposed three. The *Leishmania* mini-circle kinetoplastid, heat shock protein 70 (hsp70) and internal transcribed spacer I DNA along with human blood were detected from sibling species A only, which has not previously been considered to be a vector.

**Conclusions:**

The taxonomy of the Sri Lankan Argentipes species complex is reassessed based on the molecular data. The existence of two sibling species is proposed; sibling species A has a long sensilla chaetica (> 50% length of the second antennal flagellomere) and sibling species B has a short sensilla cheatica (< 50%). Sibling species A is incriminated as a vector for leishmaniasis in Sri Lanka.

## Background

Leishmaniasis is a health threat in as many as 88 countries [1,2]. The common forms of the disease in the old-world are cutaneous leishmaniasis (CutL), visceral leishmaniasis (VisL) [3] and post Kala-azar dermal leishmaniasis (PKDL) [4]. The disease forms are prevalent in different geographic regions. Ninety percent of previously reported VisL patients were from Bangladesh, India, Nepal, Sudan and Brazil whereas around ninety percent of the CutL cases were recorded from areas such as Afghanistan, Algeria, Brazil, Iran, Peru, Saudi Arabia and Syria [5].

Leishmaniasis was previously considered to be an exotic disease in Sri Lanka. Migrant workers returning from the Middle East were the only diagnosed patients before the early 1990’s [4]. The first case of autochthonous CutL was reported in Mamandala village of Hambantota district in 1989 [6]. Since then, the number of cases of CutL has risen and more than 2000 cases have been reported in the last decade from many parts of the country.

The parasite causing CutL in Sri Lanka has been identified as *Leishmania donovani* zymodeme MON 37 [7]. *Leishmania donovani* in the Donovani species complex is generally associated with VisL and PKDin India and Afrotropical regions [5,8]. The suspected vector(s) in Sri Lanka are sandflies from the *Phlebotomus argentipes* sensu lato Annandale & Brunette, 1908 species complex, which are known to be the vector for *Leishmania donovani* in India [9].

The occurrence of sibling or cryptic species among insect vectors that have overlapping morphological characters is very well documented. The inability to distinguish these sibling species from one other using standard morphological analysis has led to the use of biochemical tools in other organisms such as *Anopheles* mosquitoes. These include allozyme analysis and polytene chromosome banding patterns [10,11]. More recently DNA sequence analysis of conserved regions of Ribosomal DNA (rDNA) or mitochondrial markers (e.g. cytochrome oxidase subunit I or cytochrome b oxidase) have been used. In many cases, only one or two sibling species within a species complex have vectorial capacity [12]. Correct identification of vector sibling species is important as failure to do so may conceal the actual transmission pattern of the disease and will result in inadequate vector/disease control strategy. This has been well demonstrated in cases such as the differential insecticidal resistance observed among sympatric sibling species in *Anopheles* mosquitoes by [13,14]. Different micro geographic forms of *Phlebotomus papatasi* in Sudan and Egypt are suspected to play different role in leishmaniasis epidemiology [15].

In this study, different methods are used to characterize the relationships between flies within the *Phlebotomus* (*Euphlebotomus*) *argentipes* species complex in Sri Lanka and to establish their potential to be vectors for *Leishmania*.

## Methods

### Sandfly collection and identification

**Figure 1:**
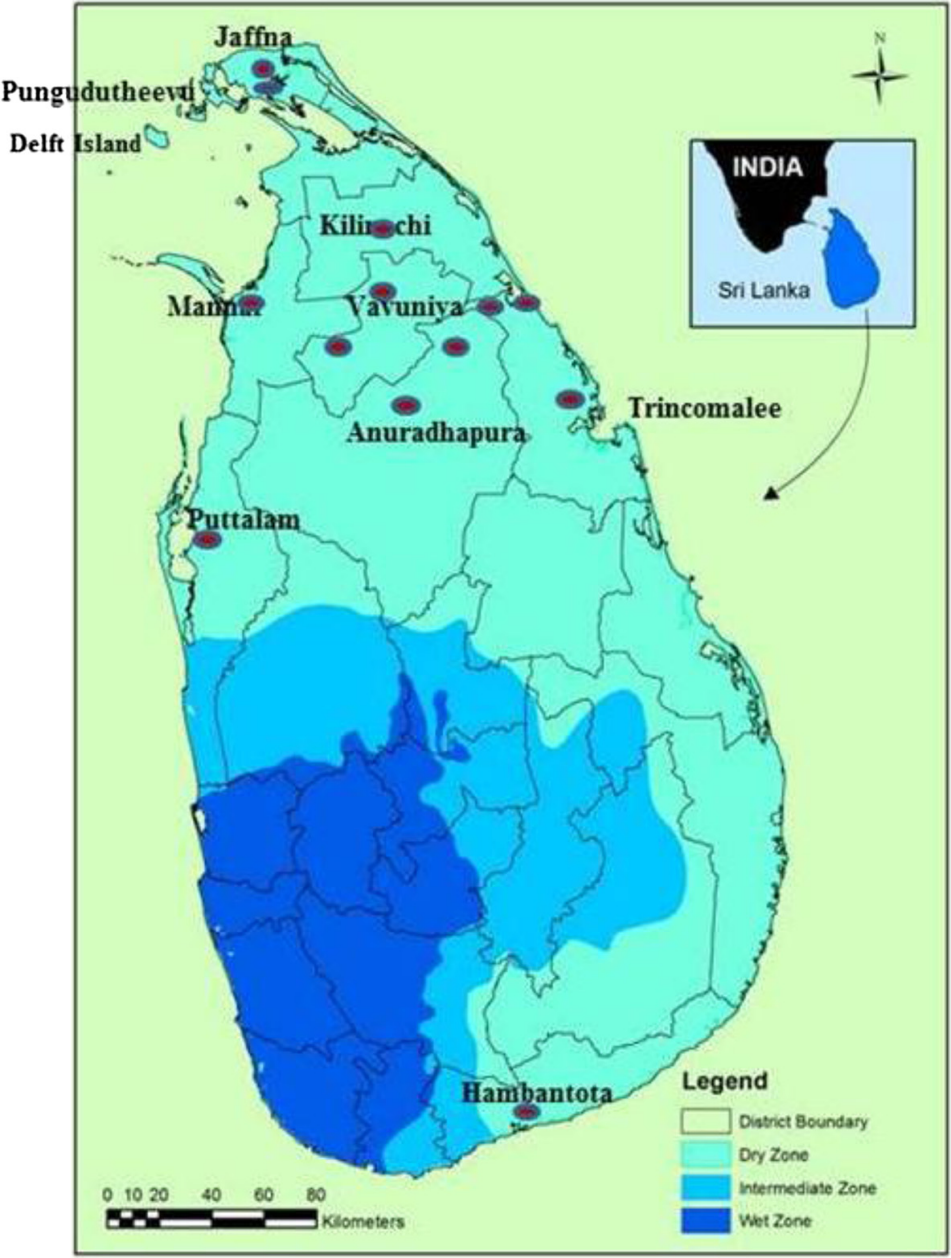
Sampling locations (marked with spots) in several districts of Sri Lanka.

Sandflies were collected between 2009–2012 from a range of sampling sites (Figure 1) using light traps, cattle-baited traps and with mouth aspirators in and around the houses. The collected flies were preserved in 70% ethanol and were identified on the basis of morphometric and meristic characters [16,17]. The wing index (R_2_/R_2+3_), wing overlap (R_1_ overlap/R_2_) and the ratios between wing length and width, second sensilla cheatica (SCII) and lengths of antennal flagellomere (AF); AFII, (AFII + AFIII) and AFI, genital coxite and genital style, genital pump and aedegal filament, lengths of head and eye, length thoracic appendage segments, and maxillary palp segments were measured using an ocular micrometer attached to an Olympus BX51 (Tokyo) microscope. The Argentipes complex was identified based on the description of Ilango [17].

DNA of the individual flies was extracted by the ethanol precipitation method. Individual flies were homogenized in extraction buffer (Tris base (pH 8.00), NaCl, SDS and EDTA) and proteinase K (4:1). 5M NaCl was added after the overnight incubation at 56°C. The supernatant was separated after centrifuging at 14000 rpm for 5 minutes. 400 μl of cold, 70% ethanol was added to the supernatant. The solution was incubated at -20°C for 1 hour. The solution was centrifuged at 14000 rpm for 30 minutes. Ethanol was removed and the pellet of DNA was washed with cold 70% ethanol. After the removal of ethanol and drying, the pellet was re-suspended with 120 μl of double distilled water.

Specific primers were used to amplify the D3 region of the large subunit ribosomal RNA gene [18] and a section of the 18S small subunit rDNA gene [19] in addition to a variable region of the mitochondrial cytochrome oxidase I (COI) gene [20]. The PCR reaction mixture was prepared as follows: 1X Taq polymerase buffer, 1.5 mM MgCl_2_, 200 μM dNTPs and 0.625U Taq polymerase (Promega, USA). 0.125 μM (for D3) and 0.6125 μM primers were used in the PCR. The reaction conditions were; 94°C of initial denaturation followed by 94°C (30s), 55°C (45s) and 72°C (1 min.) for 35 cycles and 72°C for 10 minutes for D3. 18S rDNA was amplified using the conditions described by Surendran *et al*. [19]. The cytochrome oxidase subunit I gene was amplified with an initial denaturation of 94°C (10 min.) and 40 cycles of 94°C (30s), 50°C (30s) and 72°C (1 min.) and a final extension at 72°C for 10 minutes. The cytochrome oxidase b gene was amplified with degenerate primers as previously described [21]. 2.0 mM MgCl_2_, 1.0U Taq DNA polymerase, 0.1 μM of each primer, 200 μM of dNTPs, 1X Taq buffer and 4 μl of genomic DNA was used in a total volume of 40 μl. The PCR reaction was performed with the initial denaturation of 95°C for 5 minutes, followed by 10 cycles of 94°C for 30s, 42°C for 30s and 72°C for 1.5 minutes. Another 30 cycles of 94°C for 30s, 49°C for 30s and 72°C for 1.5 minutes was followed by the final extension of 72°C for 10 minutes. Universal ITS2 primers [22] were used to amplify and sequence the ITS2 region. The PCR was performed in a total volume of 40 μl with 1x Taq Buffer, 1U Taq DNA polymerase, 200 μM dNTPs, 2.0-2.5 mM MgCl_2_ (depending on template DNA quality), 100pmoles of each primer and 4 μl of DNA. The amplification was done with an initial denaturation temperature of 95°C for 5 minutes followed by 35 cycles of 95°C for 30s, 55°C for 45s and 72°C for 1 minute. The final extension time was set at 72°C for 10 minutes.

The PCR products were sent to M/s Macrogen, South Korea for sequencing. The sequences were edited in Bioedit (v7.1.3) and aligned and deposited in Genbank (18s- Gen Bank: KC791427, KC791428, KC791429 and KC791430; D3- Genbank: JF312867, JF312868 and JF312869; CO I- Genbank: KC791430, KC791431, KC791432, KC791433, KC791434, KC791435, KC791436, KC791437 cytb- Genbank: KF416345- KF416353; ITS2- Genbank: KF416354- KF416362).

### Identification of blood meal sources

A modified precipitation test based on the micro – capillary technique was used to determine the source of the blood meal [23]. 50 μl of diluted sandfly blood meal (in 200 μl saline water) was taken into a micro Hematocrit tubes with 50 μl of human or bovine antiserum (Sigma-Aldrich- separately diluted to 1:1000). Formation of a “ring” at the interface of the two phases (blood and antiserum) indicates that the blood meal contains human or bovine tissue. A PCR based assay was performed using universal cytochrome oxidase b primers as described by Ravasan *et al.*[24] with human and cattle DNA, which are two likely hosts upon which the sandflies have fed, as positive PCR controls.

### Detection of *Leishmania* in sandflies

Sandflies collected by indoor resting collections were dissected and examined for the presence of *Leishmania* parasites.The DNA of individual flies was extracted using the method described above. A semi nested PCR was performed in Applied Bio systems 9700 thermal cycler with one common forward primer LINR4 5′(GGG GTT GGT GTA AAA TAG GG-3′) and two reverse primers; LIN17 (reverse) (5′-TTT GAA CGG GAT TTC TG-3′), and LIN19 (reverse) (5′-CAG AAC GCC CCT ACC CG-3′) as described by Aransay *et al*. [25]. The ITS1 region of *Leishmania* was amplified with the primers LITSR and L5.8S Schonian *et al*. [26]. A 600 bp region of the heat shock protein gene was amplified using the primers and method described by Garcia *et al*. [27]. A negative control was performed using DNA extracted from a male fly collected from a non-*Leishmania* endemic locality. The PCR product was visualized in 1.5% agarose gel stained with ethidium bromide. Sequencing was done by Macrogen, Europe.

The collection was carried out with the approval from the Department of Wild life Conservation, Sri Lanka and health authorities of relevant sampling sites in accordance with all the local rules and regulations regarding collection of sand flies. The study was approved by the Research committee of the Faculty of Graduate Studies, University of Jaffna.

## Results and discussion

### Sandfly collection and identification

Sandflies were collected in all the districts except Kilinochi and Mannar (Figure 1). *Phlebotomus argentipes* sensu lato was the predominant species in most districts except Trincomalee and Vavuniya (Table 1). Most of the sandfly collection points were associated with the presence of domesticated animals such as dogs and cattle.

**Table 1:** Collection detail of sandflies from different localities

**Location**	**No. of *Phlebotomus***		**No. of*Sergentomyia***	**Total**
	***Ph. (Eup.) argentipes***	**Other *Phlebotomus***		
Delft Island	1180	00	01	1181
Pungudutheevu	210	00	00	210
Chundikuli (Jaffna)	472	00	03	475
Chunnakam (Jaffna)	654	00	06	660
Kilinochi	00	00	00	00
Vavuniya	00	01	31	32
Mannar	00	00	00	00
Trincomalee	00	00	45	45
Anuradhapura	112	00	30	142
Putalam	201	00	01	202
Hambantota	487	00	03	490
**TOTAL**	**3316**	**01**	**120**	**3437**

The Argentipes complex was earlier described as a species complex with two morpho species namely A and B. Morphospecies B, which has a small sensilla cheatica in the second antennal flagellomere (the ratio of the length of the sensilla cheatica / the length of antennal flagellomere is less than 0.5) was considered as the vector for *Leishmania donovani* in South India [9]. The taxonomy of this group was reassessed by Ilango [17], based on the morphometric and meristic characters, in which the species complex was described as a group of three sibling species. All the three members, namely *Phlebotomus (Eup.) glaucus, Ph. (Eup.) argentipes* sensu stricto and *Phlebotomus (Eup.) annandalei,* described by Ilango [17] were recorded in the present collection. *Ph. (Eup.) glaucus* (females with longer sensilla cheatica in the second antennal flagellomere and males with small genital coxite compared to genital style) was found to be the dominant species.

Sequences of the 18SrDNA and D3 regions of the sibling species of *Phlebotomus argentipes* sensu lato, (with at least 10 individuals sequenced from each sibling species), did not show any sequence variation within the group.

Cytochrome oxidase subunit I and cytochrome b oxidase sequences were, however, variable within the species complex. The amino acid sequences were checked against the reference sequence, which has been reported from other taxa [28], and all were found to be coding thus ruling out the presence of any nuclear pseudo genes in the sequences.

**Figure 2:**
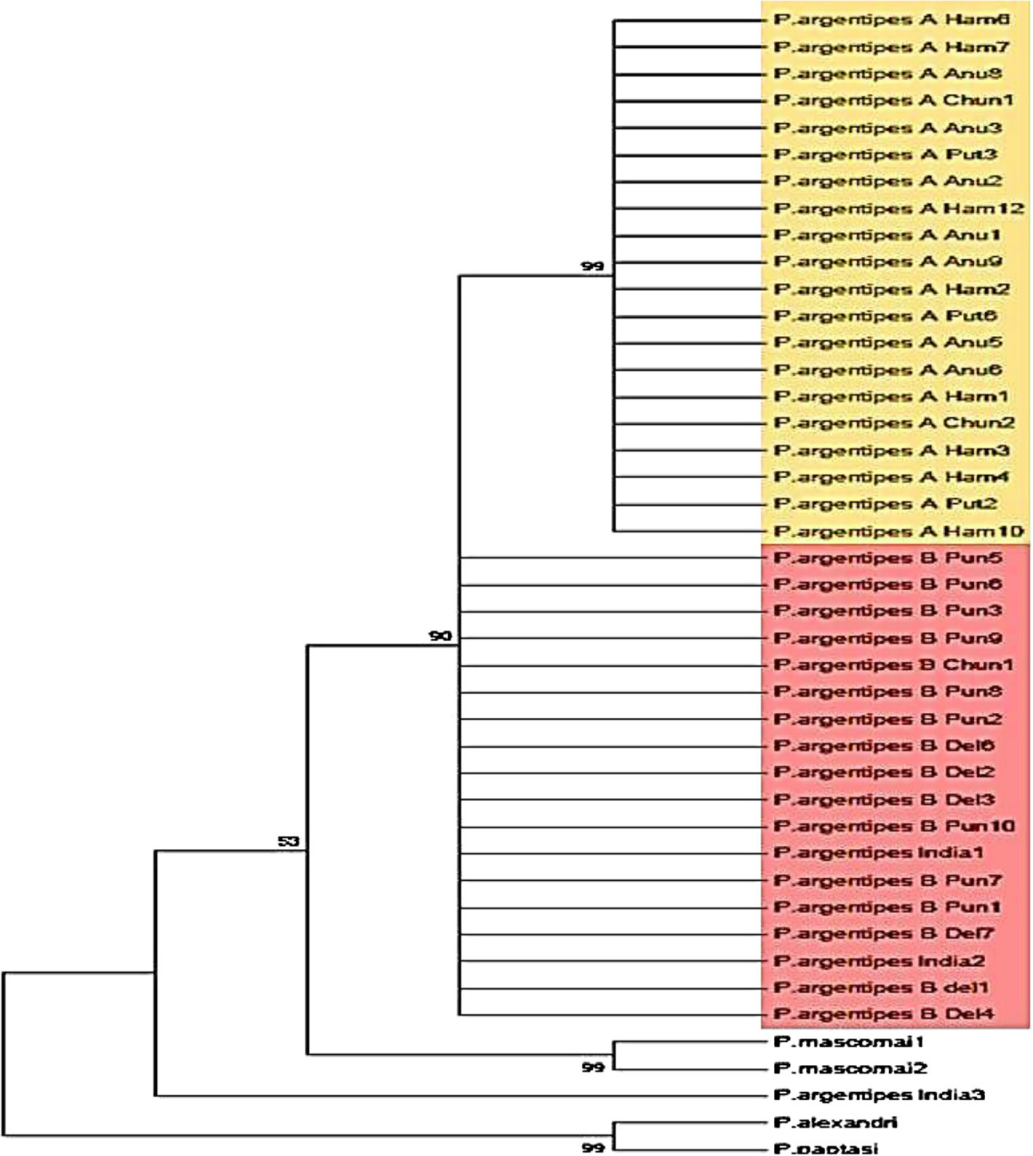
**Evolutionary relationships of the tested members of the Argentipes complex.** Tree was constructed using the Maximum likelihood method using cytochrome oxidase subunit I (HKY model with estimated gamma distribution and with 100 non parametric bootstraps) inferred by Phyml3.0 (Legends for Sri Lankan samples: Del- Delft Island, Chun- Chunnakam, Anu- Anuradhapura, Pun- Pungudutheevu, Put- Putalam).

Substitution model selection for phylogenetic tree construction was performed using the lowest Bayesian Information Criterion value. The Hasegawa, Kishino and Yano (HKY) substitution model with a gamma distribution (+G) was selected. A phylogeny (maximum likelihood) constructed from the Cytochrome oxidase subunit I sequence data with 4 classes of variable sites and the HKY + G model of nucleotide substitution (Phyml 3.0; [29]) contained one strongly supported clade (bootstrap value of 99%, Figure 2), This clade contained only those flies (males and females) with long sensilla cheatica in the second antennal flagellomere (greater than 0.50 in length compared to the length of the antennal flagellomere). All flies outside this clade had short sensilla cheatica (>0.50). This topology is not in accordance with the classification reassessment proposed by Ilango [17] on the basis of male characteristics for the Indian *Phlebotomus* (*Euphlebotomus*) *argentipes* complex. i.e. the groupings in the phylogeny based upon cytochrome oxidase sequences correspond to the sensilla cheatica ratio rather than the ratio of genital coxite/genital style. *Phlebotomus papatasi* (subgenus *Phlebotomus*) and *Phlebotomus alexandri* (subgenus *Paraphlebotomus*) were selected as out-groups along with three *Ph*. (*Eup*.) *argentipes* sensu lato deposited in Genbank. One fly (ARGIND3) identified as *Ph*. (*Eup*.) *argentipes* in the Indian Argentipes complex did not belong to the Sri Lankan species complex.

**Figure 3:**
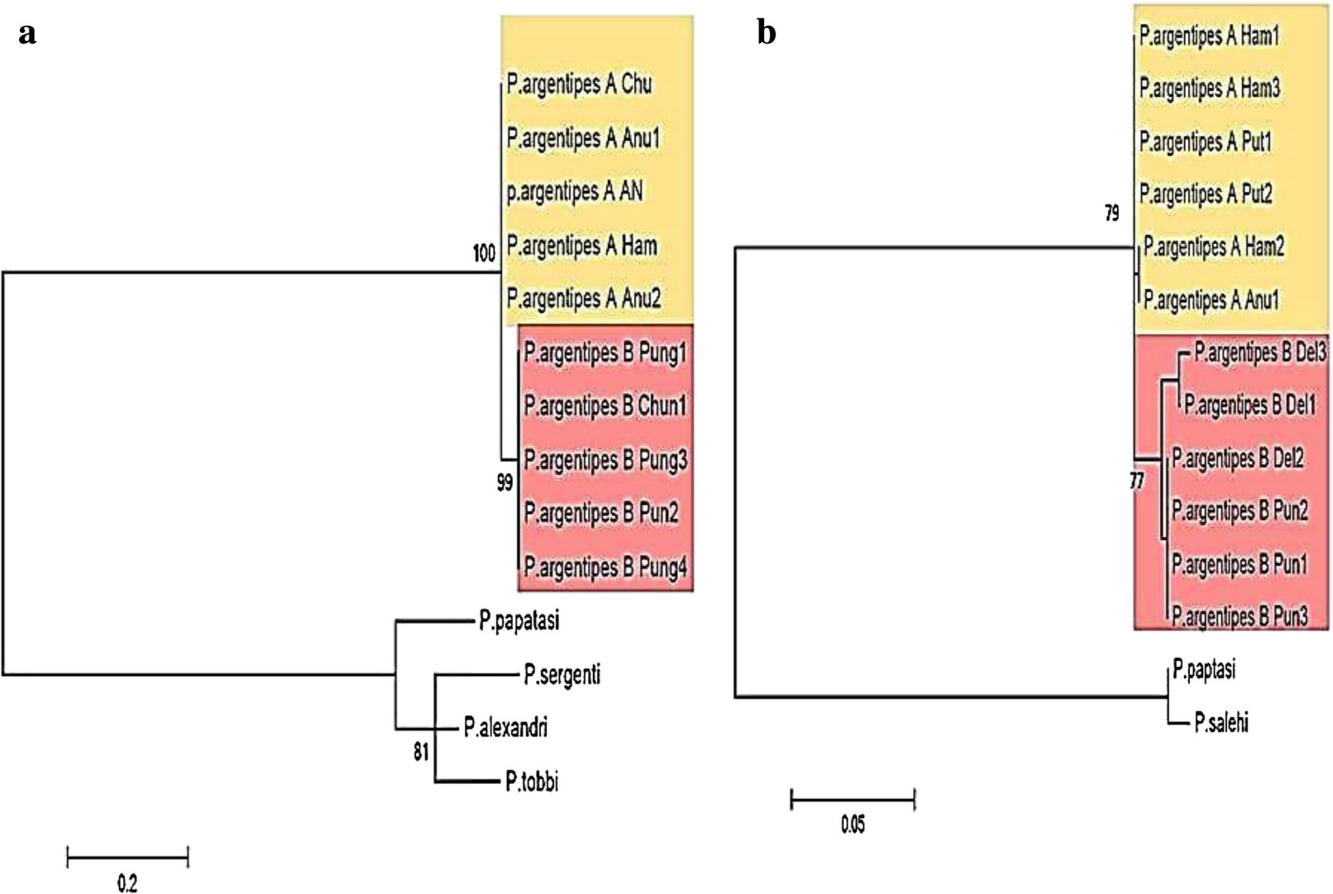
**Evolutionary relationships of the Argentipes complex using a) Cytochrome b oxidase and b) ITS2 sequences.** Trees were constructed using the Maximum likelihood method using cytochrome b oxidase gene (HKY model with estimated gamma distribution and with 100 non parametric bootstraps) inferred by Phyml3.0 (Legends for Sri Lankan samples: Del- Delft Island, Chun- Chunnakam, Anu- Anuradhapura, Pun- Pungudutheevu).

Phylogenies were also constructed from the cytochrome b oxidase and ITS2 sequence data using the same parameters and models as were used for cytochrome oxidase subunit I (Figure 3). The topology of these two trees was consistent with that for cytochrome oxidase with strong bootstrap support (>70%) for the separation of a clade, containing flies with short sensilla cheatica, from the rest.

*Ph*. (*Eup*.) *mascomai*[30], *Ph*. (*Phb*.) *papatasi* (Abbasi and Warburg Unpublished) and *Ph*. (*Phb*.) *alexandri* were [31] were used as an out group for CO I phylogeny construction. *Ph*. (*Phb*.) *papatasi*[32], *Ph*. (*Phb*.) *alexandri*[33], *Ph*. (*Phb*.) *tobbi* (Absravan *et al*. unpublished) and *Ph*. (*Phb*.) *sergenti* (Vaziri *et al*., unpublished) were used as out group in cytochrome b tree construction. *Ph*. (*Phb*.) *papatasi*[34] and *Ph*. (*Phb*.) *salehi*[35] were used as out groups in the creation of ITS2 phylogeny.

Uncorrected p distances were calculated for each gene region sequenced using the software MEGA 5.1 [36] with both transversion and transitions estimated as occurring at a uniform rate. The p distance for the ITS2 was 0.42% within each of the two distinct groups within the phylogeny and ranged from 1.3% - 2.5% between these groups. The inter-group p distance for cytochrome oxidase subunit I was 0.01% while the intra-group p distance is 1.2%-1.4%. Cytochrome b oxidase sequences had no intra-group variation but had 1.29% of inter-group species p distance. These genetic distances along with the morphological differences in the sensilla cheatica support the existence of two distinct groups within the Argentipes complex.

On the basis of our molecular data, we propose that the Sri Lankan *Phlebotomus* (*Euphlebotomus*) *argentipes* complex likely consists of only two, rather than three, sibling species. Males and females in the first of these have a length of sensilla cheatica in second antennal flagellomere/ length of second antennal flagellomere ratio of greater than or equal to 0.50 whereas males and females of the second species have a ratio of less than 0.50. With this cutoff value, the morphometrics used in the classification scheme [17] were tested with the data of 200 flies from each sibling species. Other morphometric features used in the classification, such as genital coxite/ genital style ratio, wing overlap (length of R_1_ over lap over the R_2_/ length of R_2_) and wing index (R_2_/R_2+3_) were found to be overlapping. The sensilla cheatica ratio was not over lapping with the distribution pattern of 0.33 (minimum) to 0.52 (maximum, observed in only one fly, which might be a slight error in measurement) for sibling species B and with the minimum of 0.53 to the maximum of 0.75 in sibling species A. Generally males possessed shorter sensilla than females.

Another taxonomic feature which could also be used in classification might be the size of the flies given that sibling species B is larger and wider (across the third abdominal segment) than sibling species A. Sibling species A is also darker compared to the sibling species B.

### Identification of blood meal sources

**Figure 4:**
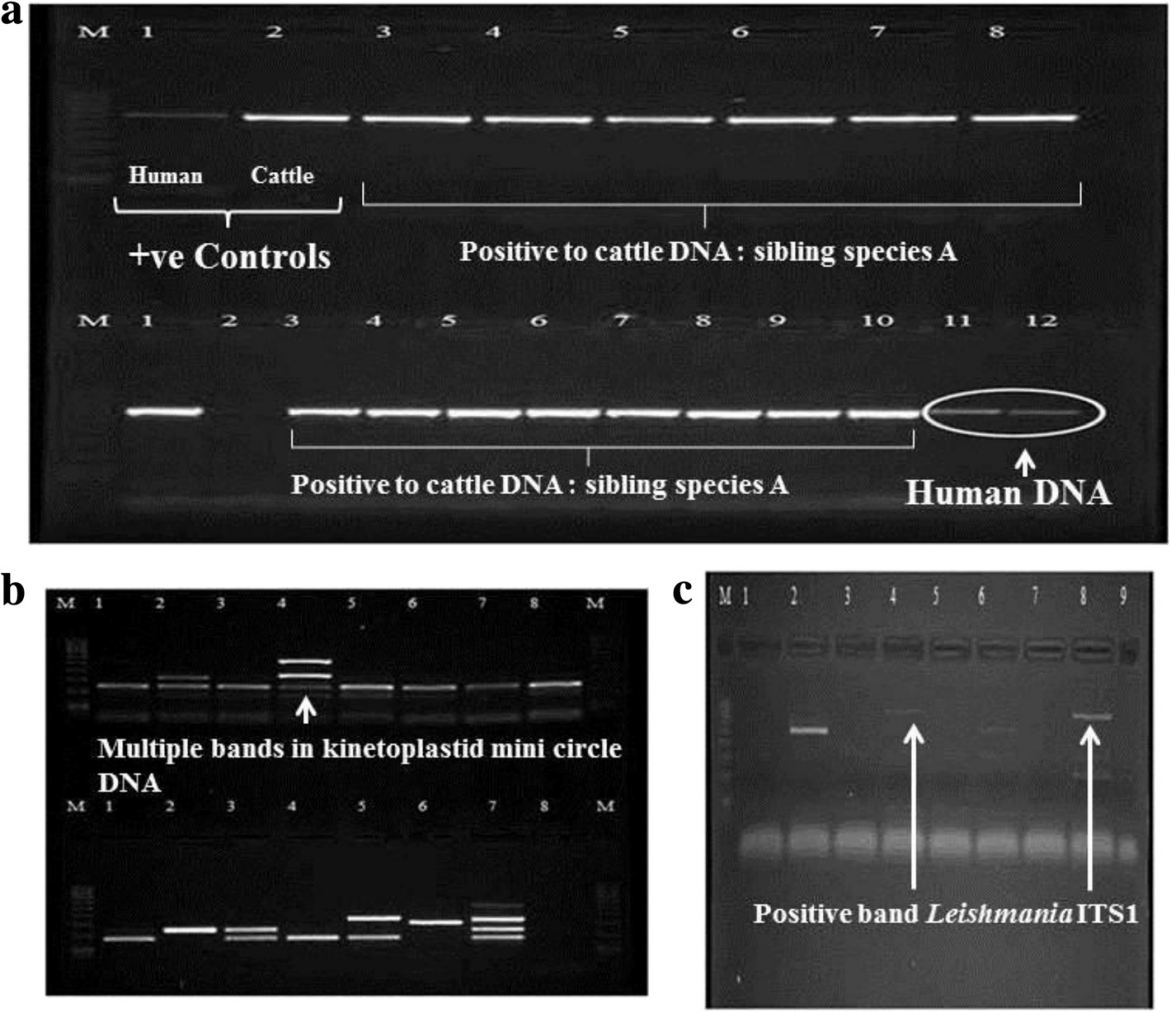
**Gel images for blood meal analysis (a) and *Leishmania *detection PCR (b). a)** Agarose gel (1.5 %) image of the blood meal analysis using the universal vertebrate cytb primers; (M- marker lane, 1- PCR done with human DNA, 2- PCR done with cattle DNA, 3-8 in row1 and 1-12 in row2 the PCR done with sandfly extractions). **b)** Semi nested PCR amplified products of mini circle kinetoplastid DNA: (M- Marker [100bp] Lanes 1-8 in row1 and 1-7 in row 2 are female *Ph. (Eup.) argentipes* sibling species A from Hambantota, lane 8- negative control; male *Ph. (Eup.) argentipes* sibling species A extract from Hambantota ). **c)** ITS1 PCR amplified products in 1.5% agarose gel (M- 100 bp marker, 1 and 9- negative controls [1- female sibling species B from Delft, 9- male sibling species A from Hambantota, all the other lanes are sibling species A female extracts from Hambantota).

Blood meal analysis of the blood-fed flies identified as sibling species A indicates that these flies may have fed on humans. A total of seventy one percent of blood-fed females (36 of a total of 51 flies) from sibling species A tested positive to human antiserum (Table 2). The gel run with positive control DNA (cattle and human) indicated the presence of human blood in four flies out of 40 randomly selected blood fed flies (Figure 4a).

**Table 2:** **Blood meal analysis results for the*Phlebotomus (Euphlebotomus) argentipes*sibling species A from Hambantota, Sri Lanka (n = 36)**

**Results**	**Percentage**
Positive to human antiserum	71
Positive to bovine antiserum	22
Negative to both	05
Positive to both	02

### Vector incrimination

**Figure 5:**
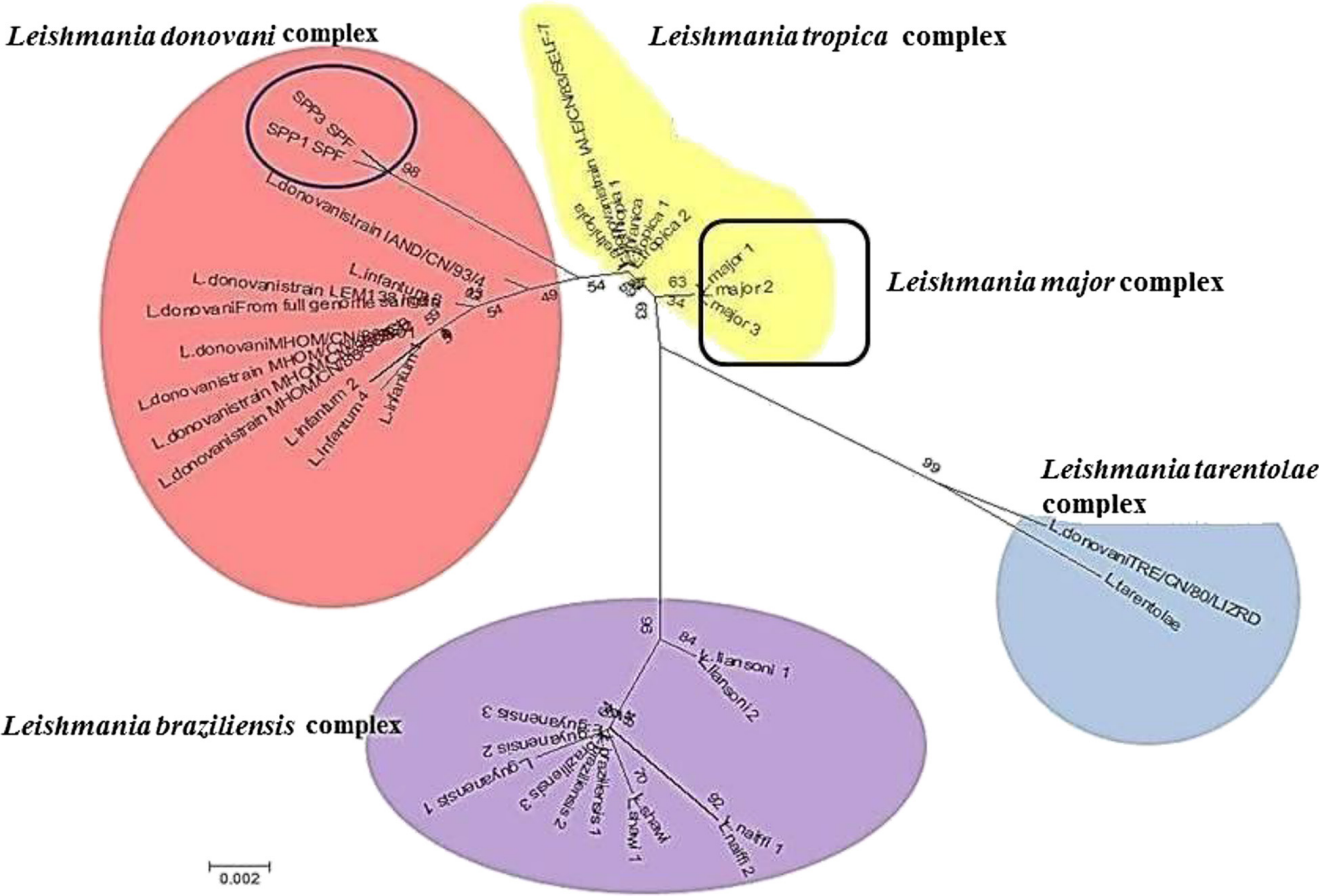
**Evolutionary relationships of the tested members of the *Leishmania *complex.** Tree was created from the Maximum likelihood method using partial sequence of heat shock protein 70 gene (HKY model with estimated gamma distribution and with 100 non parametric bootstraps) inferred by Phyml3.0 (Legends for Sri Lankan samples: SPP3 and SPP1; blue circled).

*Leishmania* minicircle kinetoplastid DNA was amplified from 65% (n = 40 randomly selected flies caught from leishmaniasis endemic village in Hambantota) of the processed females of sibling species A and B (Figure 4b). The multiple bands that were found in some samples are likely to be the amplified products of other classes of mini circle DNA. The ITS1 region, which is targeted using primers with greater specificity, was amplified to confirm the presence of *Leishmania* sp.. Typing of *Leishmania* species was done on the basis of sequencing a section of heat shock protein 70 (hsp70) gene. Partial hsp70 gene sequence (360 bp) (Genbank: KF416363 and KF416364) show that the *Leishmania* sequences obtained from *Ph. argentipes* most closely resemble those from India and that they likely to belong to the *Leishmania donovani* group (Figure 5). *Le. donovani* extracted from a lizard (Genbank: TR/CN/180/LIZRD) groups with *Le. tarentolae,* which is a parasite found in reptiles, and seems likely to have been a misidentification.

*Leishmania* DNA was present in blood fed as well as unfed females (n = 5). The presence of *Leishmania donovani* DNA in unfed individuals (as determined by the absence of any blood meal in PCR) confirm that the sibling species A, with long sensilla cheatica, is likely to be a vector for leishmaniasis in Sri-Lanka. None of the tested sibling species B (n = 42) female flies had *Leishmania* DNA within them.

## Conclusions

The combined molecular and morphological data indicate that there are likely two sibling species within the *Phlebotomus* (*Euphlebotomus*) *argentipes* complex in Sri Lanka. The sibling species that is proposed not to be a vector for *Leishmania* transmission in India seems, in contrast, likely to be a vector for cutaneous leishmaniasis and possibly visceral leishmaniasis in Sri Lanka. It will be interesting to study further evolutionary differences between these sibling species and their disease transmission potential.

## Competing interests

Authors declare that they do not have any competing interests.

## Authors’ contributions

SNS, KG and SLG conceptualized the study.KG, BSLP, PJJ and AS were involved in sampling and field work. KG and SLG were involved in laboratory analysis. KG, SLG and SNS were involved in manuscript preparation and editing. All authors approved the final version of the manuscript.
